# Comparison of the prognostic value of microscopically measured invasive width versus macroscopic width in cutaneous melanoma shows the superiority of microscopic invasive width measurement

**DOI:** 10.1111/cup.14220

**Published:** 2022-03-21

**Authors:** Mark Bamford, Louisa Udensi, Arushi Khanna, Marie O'Riordan, Gerald Saldanha

**Affiliations:** ^1^ Department of Cellular Pathology University Hospitals of Leicester NHS Trust Leicester UK; ^2^ Leicester Medical School University of Leicester Leicester UK; ^3^ Leicester Cancer Research Centre University of Leicester Leicester UK

**Keywords:** biomarker, cancer, malignant melanoma, prognosis, skin

## Abstract

**Background:**

Invasive width, the distance between the most peripheral invasive melanoma cells on the section where Breslow thickness (BT) was measured, was recently identified as a prognostic feature. It is unclear whether a routine measurement is justified, given that macroscopic width is already included in many melanoma histopathology reports and may itself be a prognostic feature. This study sought to investigate this.

**Methods:**

A retrospective cohort of 718 melanoma patients in which macroscopic width had been stated in the original histopathology report was used. Survival analysis was performed.

**Results:**

Macroscopic and invasive widths were positively correlated (*p* < 0.001). Invasive width was typically smaller than the paired macroscopic width (median difference 3.7 mm, *p* < 0.001), a difference seen across all T groups. Both macroscopic and invasive widths were significantly associated with melanoma survival in Kaplan–Meier analysis, including overall survival, but invasive width survival curves were more widely separated. Both were significantly associated with outcome after correction for BT in Cox proportional hazards regression, but the models containing invasive width had a substantially better fit.

**Conclusions:**

This study shows that both macroscopic and invasive widths have prognostic values, but confirms that the latter is superior. It supports further investigation of this feature's prognostic value.

## 1 INTRODUCTION

Malignant melanoma is a form of skin cancer with a particularly poor prognosis when the disease becomes advanced.^1^ Simple histopathologic features assessed on hematoxylin and eosin (H&E) staining are the gold standard for clinical staging. In the AJCC 8th edition TNM staging system (AJCC8), these are BT, ulceration, and microsatellitosis.^2^ In addition, features such as tumor‐infiltrating lymphocytes, regression, mitotic index, growth phase, perineural invasion, and lymphovascular invasion are deemed to have prognostic value but are not used for staging. Given the richness of features in an H&E‐stained slide, it seems that novel prognostic features could be readily identified.

To address this, enhancements of BT have been investigated. BT only measures the tumor dimension in a single plane perpendicular to the skin surface, so it may be possible to make a prognostic distinction between two melanomas with the same BT if either the density of tumor cells in the H&E section or the dimension of tumor in a different plane is considered. Thus, a melanoma with demonstrably more invasive cells directly surrounding the position where BT has been measured should, on average, have a worse outcome. This idea led to the development of the so‐called Breslow density (BD),^3^, ^4^ and greater BDs were indeed associated with poorer outcome after adjusting for BT. In a further iteration of this general theme, an improved metric was devised, namely the total area occupied by invasive melanoma cells on the same histopathology slide where the BT measurement was made. Known as the calculated tumor area (CTA), this was found to be a better prognostic feature than both BT and BD.^5^ A drawback of CTA was that it entailed the use of a subjective estimated value. To circumvent this, we assessed the use of a feature that could be measured objectively, namely the width of the area containing invasive melanoma cells. This was measured microscopically in an axis perpendicular to BT and ignored in situ cells extending peripherally.^6^ This was found to be strongly associated with outcome, even more so than BT, although not as powerful as CTA.^6^ The concept of using both BT and invasive width in perpendicular axes capturing different dimensions of tumor burden seems to be a plausible way to enhance BT and might be very acceptable to histopathologists, as both BT and invasive width need only an H&E‐stained section.

One potential argument against the use of invasive width is that the macroscopic width is often routinely recorded anyway, and maybe this makes invasive width redundant. However, macroscopic width would be expected to typically include in situ melanoma, which has no metastatic potential. Nevertheless, a head‐to‐head comparison of the prognostic value of invasive width and macroscopic width has never been tested. We anticipate that the presence of in situ disease will make invasive width superior but seek to demonstrate this objectively. Thus, this study explored the extent that invasive width measurement differed from macroscopic width and to establish their different prognostic values.

## 2 MATERIALS AND METHODS

### 2.1 Patients and width measurements

**FIGURE 1:**
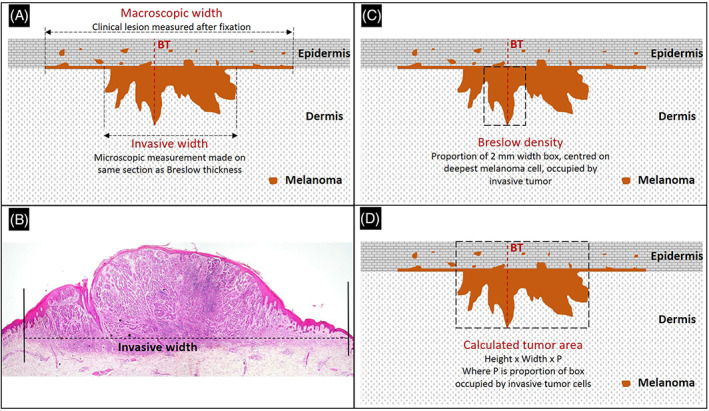
An illustration of macroscopic and invasive microscopic width measurement (A). The invasive width of an actual case is shown (B). For comparison to width, two other histopathological prognostic features developed by some of the authors are illustrated, Breslow density (C), and calculated tumor are (D). BT, Breslow thickness (12.5x magnification)

The patients in this study were a subset of those analyzed and described previously.^6^ The final population for that study comprised patients diagnosed with melanoma at the University Hospitals of Leicester between 2004 and 2014, constituting a final population of 1329. These were all primary excisions and no partial biopsy specimens were included. This study includes the subset of cases from the study of Saldanha et al.,^6^ where the macroscopic width was documented in the histopathology report. We assessed the pathology report in each case to identify either clinical or macroscopic width. The macroscopic dimension, as measured by the pathologist after fixation, was the only one consistently present and was therefore used for analysis. While the largest tumor dimension was almost always present (assumed to be the tumor length), the width was frequently omitted because this is not mandated in UK reporting guidelines,^7^ resulting in the exclusion of 611 cases for a final study population of 718 where macroscopic with was recorded. These cases were all metastasis‐free at the time of presentation. The invasive widths were measured as described.^6^ Briefly, this was the distance between the two most peripheral invasive melanoma cells in an axis approximately perpendicular to that used for BT and including any “skip” lesions between seemingly separate invasive foci. This is illustrated in Figure 1 and the other prognostic histopathological features developed by two of the authors (G.S. and M.B.) are shown for comparison.

### 2.2 Statistical analysis

Analyses were carried out using R version 3.5.2^8^ and R studio version 1.1.463,^9^ with *p* < 0.05 regarded as significant using two‐tailed tests. Baseline statistics for numerical variables consisted of the median and interquartile range (IQR) and for categorical variables, counts, and percentages. To determine the association between invasive width quartiles and BT, age, mitoses, and macroscopic width a Kruskal–Wallis test was performed. To determine the association of invasive width and the categorical variables sex, site, ulcer, microsatellites, and AJCC8 a *χ* ^2^ test was used. For the association of invasive width as a continuous variable with macroscopic width, a Spearman rank correlation test was used. To compare invasive and macroscopic width as continuous variables a Wilcoxon signed‐ranks test was used.

Survival analysis was performed for three end‐points using Cox proportional hazards (CPH) regression: overall survival (OS), melanoma‐specific survival (MSS), and metastasis‐free survival (MFS) as described previously.^6^ The Survival version 2.43‐3^10^ and survminer version 0.4.5^11^ R packages were used for CPH regression models. The proportionality assumption was checked with plots of scaled Schoenfeld residuals against transformed time and a goodness‐of‐fit test. Proportionality was not violated. CPH model fit was compared using the Bayesian information criterion (BIC).^12^

## 3 RESULTS

### 3.1 Baseline patient characteristics

In 718 cases, the median patient age was 63 years and 49.3% were female. The median BT was 0.9 mm. The baseline characteristics of the patients are shown in Table 1. They are reflective of the case mix seen in a typical UK hospital, with a majority of thin melanomas. The median invasive width and macroscopic width were 4.2 (IQR: 2.0–7.5) and 8.0 mm (IQR: 6.0–12.0) respectively. The invasive widths ranged from 0.1 to 45 mm and macroscopic width from 1.0 to 55 mm. Age, site, BT, ulcer, mitotic index, microsatellites, and AJCC8 stage were all significantly associated with invasive width after it was categorized into quartiles, also shown in Table 1. The breakpoints of invasive width quartiles were 1.8, 4.1, and 7.4 mm.

**TABLE 1:** Baseline statistics for patients included in this study

	Invasive width					
*n*	Overall	Quartile 1	Quartile 2	Quartile 3	Quartile 4	*p* value
	718	164	194	176	184	
Age (median [IQR])	63 [50, 73]	59 [46, 69]	57 [47, 71]	63 [51, 73]	70 [60, 79]	<0.001
Sex (male, %)	364 (50.7)	81 (49.4)	93 (47.9)	88 (50.0)	102 (55.4)	0.496
Site (%)						0.027
Acral	18 (2.5)	2 (1.2)	2 (1.0)	4 (2.3)	10 (5.4)	
H + N	115 (16.0)	24 (14.6)	31 (16.0)	25 (14.2)	35 (19.0)	
Lower limb	180 (25.1)	31 (18.9)	52 (26.8)	43 (24.4)	54 (29.3)	
Trunk	251 (35.0)	61 (37.2)	66 (34.0)	67 (38.1)	57 (31.0)	
Upper limb	154 (21.4)	46 (28.0)	43 (22.2)	37 (21.0)	28 (15.2)	
BT (median [IQR])	0.9 [0.5, 2.3]	0.4 [0.3, 0.6]	0.8 [0.6, 1.1]	1.2 [0.8, 2.1]	4.2 [2.2, 7.0]	<0.001
Ulcer (yes, %)	140 (19.5)	2 (1.2)	10 (5.2)	25 (14.2)	103 (56.0)	<0.001
Mitoses (median [IQR])	1 [0, 3]	0 [0, 0]	1.00 [0.00, 2.00]	1.00 [0.00, 3.00]	5.00 [2.00, 10.00]	<0.001
Microsatellites (yes, %)	21 (2.9)	0 (0.0)	2 (1.0)	2 (1.1)	17 (9.2)	<0.001
Invasive width (median [IQR])	4.2 [2.0, 7.5]	1.1 [0.3, 1.5]	3.0 [2.4, 3.5]	5.5 [4.9, 6.5]	11.0 [8.7, 15.9]	NA
Macroscopic width (median [IQR])	8.0 [6.0, 12.0]	7.0 [5.0, 9.3]	7.0 [5.0, 9.0]	8.0 [7.0, 11.0]	14.0 [10.0, 20.0]	<0.001
AJCC 8 (%)						<0.001
IA	273 (38.0)	146 (89.0)	87 (44.8)	34 (19.3)	6 (3.3)	
IB	227 (31.6)	18 (11.0)	92 (47.4)	88 (50.0)	29 (15.8)	
IIA	68 (9.5)	0 (0.0)	9 (4.6)	32 (18.2)	27 (14.7)	
IIB	57 (7.9)	0 (0.0)	3 (1.5)	15 (8.5)	39 (21.2)	
IIC	72 (10.0)	0 (0.0)	1 (0.5)	5 (2.8)	66 (35.9)	
III	21 (2.9)	0 (0.0)	2 (1.0)	2 (1.1)	17 (9.2)	

Abbreviations: BT, Breslow thickness; H + N, head and neck; IQR, interquartile range; NA, not applicable.

### 3.2 Relationship between invasive width and macroscopic width

**FIGURE 2:**
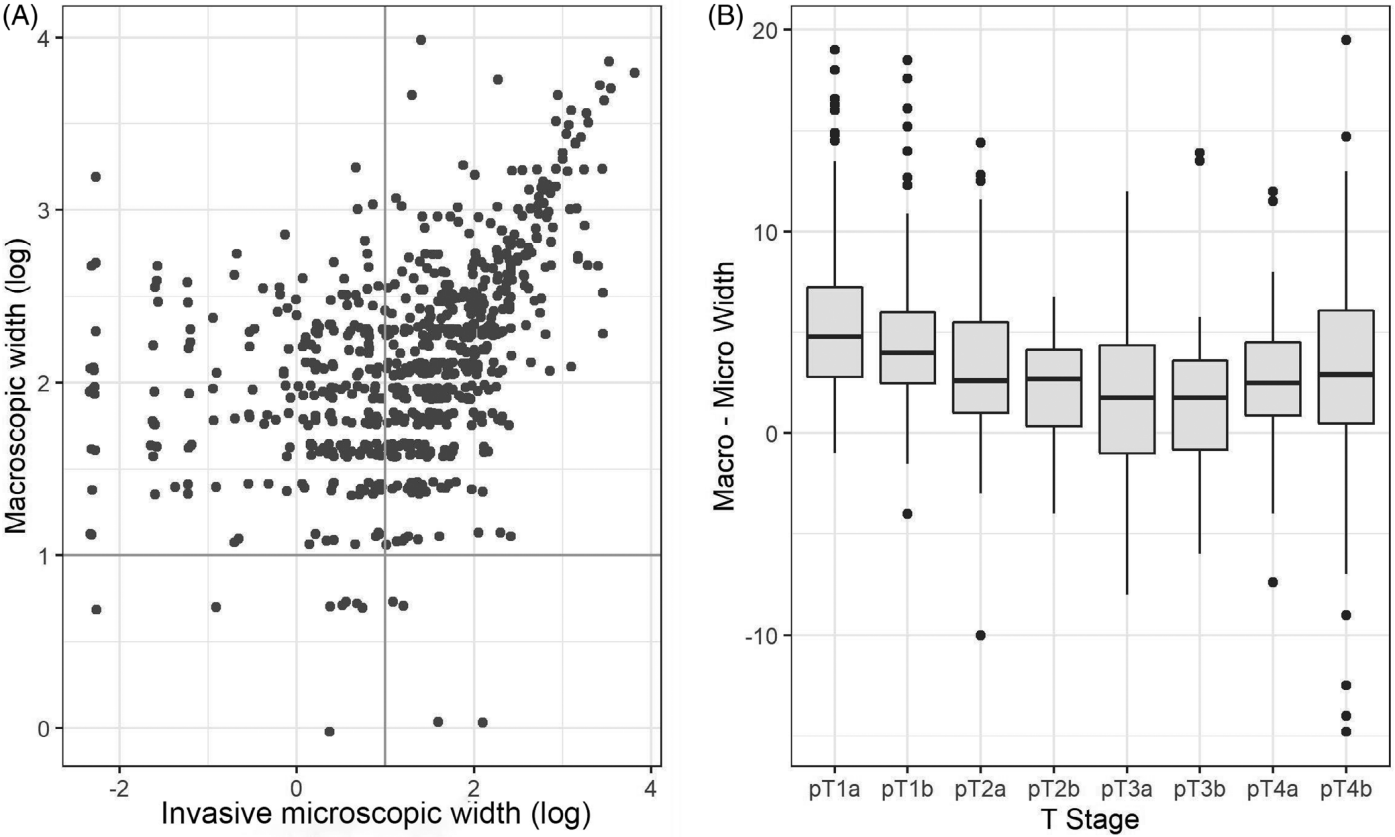
Scatter plot showing microscopically measured invasive width and macroscopic width. A natural log scale is used purely for display purposes to better see right‐skewed values. The lines in *x* and *y* show log‐scale values of 1 (A). A plot of T group and the within sample difference between macroscopic and microscopic width is shown (B)

In a cross tabulation of macroscopic and invasive widths, the median macroscopic width was the same for each lower two quartiles of invasive width, namely 7.0 mm, and thereafter increased to 8.0 mm, and then 14 mm for the third and fourth quartiles. This relationship was significant (*p* < 0.001, Table 1). This is further shown in the scatter plot in Figure 2A, in which paired values are plotted. A log scale was used for easier visualization of right‐skewed values. The Spearman correlation coefficient was 0.51, *p* < 0.001. The x‐ and y‐axis log‐scale values of 1.0, which is 2.72 mm, are shown by lines that split the plot into four quadrants. The points at low values of invasive width are preferentially in the upper left quadrant, in keeping with macroscopic width being systematically higher than invasive width, particularly at low values. Indeed, the median difference of macroscopic width versus invasive width for paired measurements is 3.7 mm (IQR: 5 mm), consistent with a macroscopic width being systematically higher (*p* < 0.001). This is further demonstrated in the boxplot shown in Figure 2B, where the paired differences between invasive and macroscopic widths are shown for each T group. It is worth noting that the median paired difference is above zero for every T stage. In summary, these data show that macroscopic and invasive widths are significantly correlated but that macroscopic width tends to be higher and in some cases is substantially higher.

### 3.3 Invasive width, macroscopic width, and melanoma outcome

**FIGURE 3:**
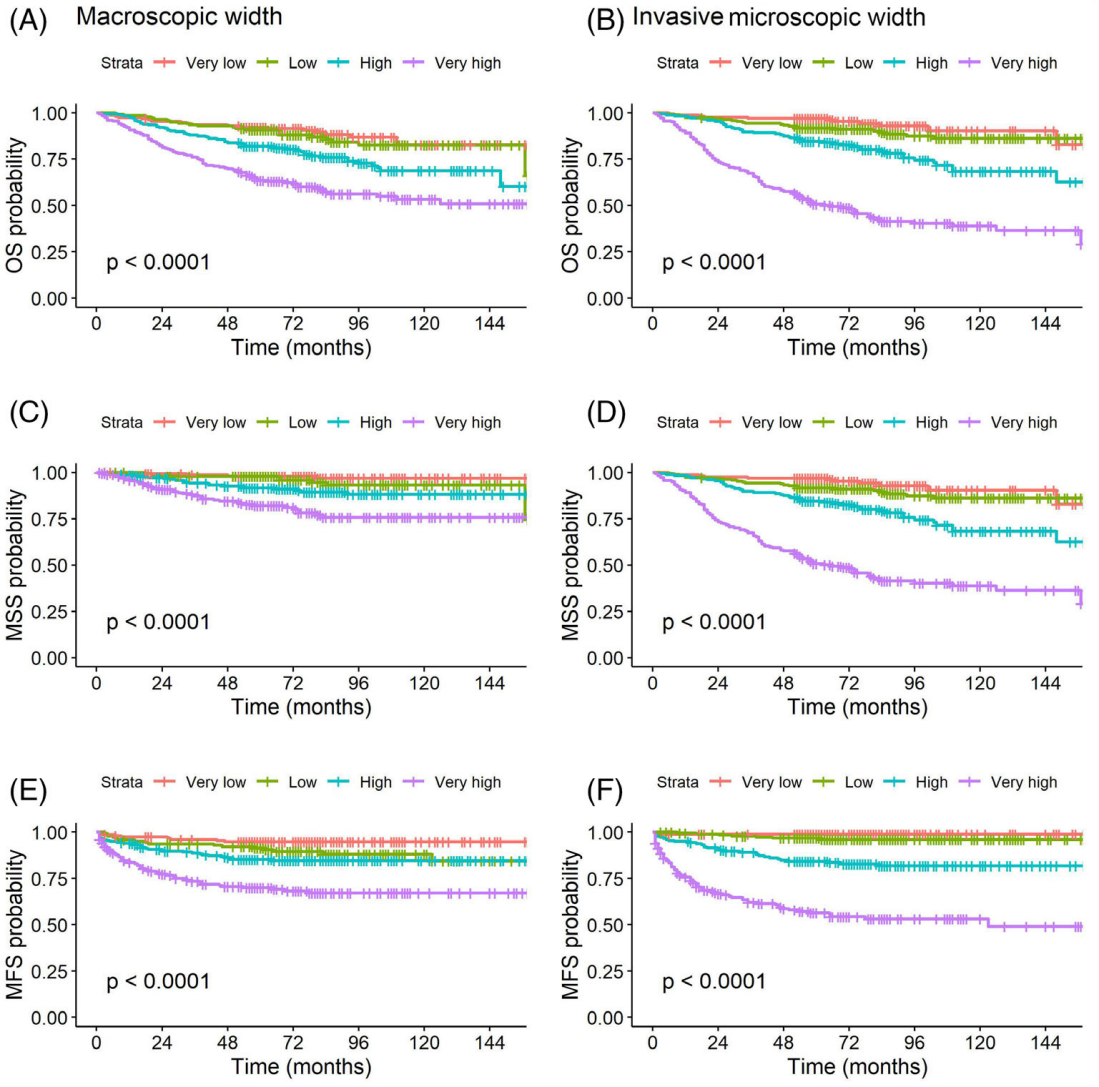
Kaplan–Meier plots for microscopically measured invasive width and macroscopic width. Macroscopic width quartile plots for OS, MSS, and MFS (A, C, and E) and invasive width quartile plots for OS, MSS, and MFS are shown (B, D, and F)

Invasive width and macroscopic width were each categorized into quartiles, then Kaplan–Meier plots were made to compare their univariable effect on three outcomes: OS, MSS, and MFS. The results are shown in Figure 3.

For each outcome, all quartile group curves follow a pattern of increasing width being associated with poorer outcome but notably the curves for invasive width are further apart overall, suggesting that it may explain more variation in outcome than macroscopic width. In support of this, the univariate HR for macroscopic width quartiles in CPH regression for OS, MSS, and MFS had a range from the lowest (1.0 by definition for each) to the highest quartile of 4.3, 9.3, and 7.3, respectively. The values for invasive width were 11.9, 64.5, and 51.0.

To compare macroscopic and invasive widths in a more clinically relevant manner, they were included in a CPH model for all three outcomes with their effects on outcome adjusted for the gold standard histopathologic prognostic feature, BT. The macroscopic width had prognostic significance for all three outcomes but the HR was not as high as BT. In contrast, the invasive width was also significant for each outcome but had a higher HR than BT and made the adjusted HR for BT non‐significant for MSS and MFS. These data are shown in Table 2. To further assess the value of macroscopic and invasive widths after correcting for the effect of BT, the model BICs were assessed. The models with invasive width had a consistently lower BIC than the equivalent models with macroscopic width, in keeping with better explanatory ability for invasive width. The difference in BIC was by more than 10 points, which indicates a substantial difference in explanatory ability.^12^ These findings are shown in Table 3.

**TABLE 2:** Cox proportional hazards models for BT, macroscopic width, and invasive width

Model 1	HR (95% CI)	Model 2	HR (95% CI)
OS			
BT	1.11 (1.08‐1.14, *p* < 0.001)	BT	1.04 (1.00–1.08, *p* = 0.034)
Macro width	1.04 (1.02–1.06, *p* < 0.001)	Invasive width	1.09 (1.06–1.12, *p* < 0.001)
MS			
BT	1.12 (1.08–1.16, *p* < 0.001)	BT	1.03 (0.98–1.09, *p* = 0.289)
Macro width	1.04 (1.01–1.07, *p* = 0.002)	Invasive width	1.11 (1.08–1.15, *p* < 0.001)
MFS			
BT	1.11 (1.08–1.14, *p* < 0.001)	BT	1.02 (0.98–1.06, *p* = 0.393)
Macro width	1.03 (1.01–1.05, *p* = 0.004)	Invasive width	1.11 (1.08–1.14, *p* < 0.001)

*Note*: Model 1 contained BT and macroscopic width with three outcomes. Model 2 contained BT and invasive width for the same outcomes.

Abbreviations: BT, Breslow thickness; CI, confidence interval; MFS, metastasis‐free survival; MSS, melanoma specific survival; OS, overall survival.

**TABLE 3:** Comparison of model BICs

	Macroscopic width	Invasive width	Difference
OS	2179.9	2153.4	26.5
MSS	857.0	831.5	25.5
MFS	1436.5	1399.5	37.0

*Note*: These models also all contained Breslow thickness.

Abbreviations: MFS, metastasis‐free survival; MSS, melanoma specific survival; OS, overall survival.

## 4 DISCUSSION

Invasive melanoma width has previously been shown to have a significant association with outcome^6^ but no one has objectively shown whether this microscopically measured prognostic feature has any advantage over macroscopic width. This is important because macroscopic width is already measured routinely at many centers and is conceptually very similar to invasive width, so there needs to be evidence‐based justification for the additional effort of measuring this new feature. Here, we confirm that both measurements are significantly correlated but macroscopic width tends to be larger, which is expected because it includes in situ disease. We also show that both macroscopic and invasive widths have a significant association with melanoma outcome but invasive width appears to explain outcome best.

This dataset is a subset of that used to investigate invasive width previously,^6^ including only those cases where we were able to identify macroscopic width. Despite this, the dataset was sufficiently large to address the main research question and Table 1 shows that the sample was broadly representative of UK melanoma cases. Importantly, this study analyzed three outcomes, thus providing strong internal validation of the findings. One important outcome that was missing was sentinel node status, but this was not routinely measured at the research setting at the time the cases were diagnosed.

Cases where invasive width was greater than macroscopic width are likely to be due to situations where the dermal and subcutaneous growth undermined any surface in‐situ disease, especially pT3 and pT4 lesions, as can be seen in Figure 1B. The demonstration that macroscopic width and invasive width are different in both absolute size and prognostic value may seem rather trivial to investigate but objective demonstration is important because some investigators have indeed used macroscopic rather than invasive width in prognostic models,^13^, ^14^, ^15^ presumably because macroscopic width is readily available in many histopathology reports. If these same investigators had used invasive width, they might have had different and arguably better results. In this study, we did not make comparisons to CTA, as this was done in our previous investigation of invasive width.^6^

This study and the previous analysis of invasive width^6^ provide early support for further investigation of this metric, which has similarities to BT. In particular, both are surrogates of tumor burden, but are complementary as they are measured in perpendicular axes. If further studies are supportive, it would be easy to capture routinely in melanoma reports, which is a prerequisite for its use in large‐scale studies of validity and potential use in future prognostic algorithms.

The cases in this study overlap with those used before. BD was assessed using 100 sequential cases from the pathology archives in Leicester from January 1, 2004.^3^ The results were subsequently validated^4^ by extending the number to 970 plus 359 melanomas from another center, Nottingham. CTA analysis used the same cases, which was considered scientifically valid because this was a brand new feature. However, more cases needed to be excluded because of the particular requirements of CTA measurement, leaving 918 Leicester and 321 Nottingham cases. Microscopic invasive width analysis^6^ also used these same Leicester cases, but they were extended to 1329. Nottingham cases were not used. An overlap in used cases was again regarded as scientifically valid because this was another new feature. In this study, the cases were entirely a subset of Saldanha et al.,^6^ but comprising only those where macroscopic width could be found in the pathology report. This required manual review of 1329 free‐text pathology reports, which was laborious and time consuming, hence these data were not available for inclusion in Saldanha et al.^6^

Invasive width and BT could be used to create a composite measure. Indeed this was addressed in our previous publication,^6^ finding that together width and BT would overestimate risk in wide and deep melanomas unless an interaction was accounted for. Moving forward, we believe that microscopic invasive width is so simple and conceptually similar to BT that it may have the best chance for translation into practice. Ultimately, this will require external validation and if sufficient evidence of prognostic value were to accrue, it might become a strong candidate for routine measurement in melanoma reports, at which point microscopic width would be available on thousands of cases for definitive comparison to established prognostic features.

In conclusion, this study shows that invasive width is a better prognostic feature than macroscopic width. Crucially, while macroscopic width is simple to measure at the time of specimen dissection, it cannot serve as a like‐for‐like replacement for invasive width. These findings support wider investigation of invasive width.

## CONFLICTS OF INTEREST

The authors declare no conflicts of interest.

## ETHICS STATEMENT

The use of human tissue was approved by an NHS REC (17/EM/0125) on April 20, 2017 and with exemption from consent.

## Data Availability

The data that support the findings of this study are available from the corresponding author upon reasonable request.
